# Effect of Al Content on Corrosion Properties in Die-Cast AZXW Alloys

**DOI:** 10.3390/ma19132760

**Published:** 2026-06-29

**Authors:** Hongxiu Liu, Bong-Sun You, Jun-Ho Bae, Jae-Yeon Kim

**Affiliations:** 1 School of Materials Science and Engineering, Changwon National University, 20 Changwondaehak-ro, Uichang-gu, Changwon 51140, Gyeongsangnam-do, Republic of Korea; halolhx@kims.re.kr; 2 Lightweight Materials Research Division, Korea Institute of Materials Science, 797 Changwondae-ro, Seongsan-gu, Changwon 51508, Gyeongsangnam-do, Republic of Korea; bsyou@kims.re.kr

**Keywords:** AZXW alloy, Al content, corrosion properties

## Abstract

This study evaluates the corrosion behavior of high-pressure die-casting AZXW alloys, focusing on the influence of Al content (6, 9, 11, and 13 wt.%). The Al effect on corrosion resistance is characterized by a dual effect. The corrosion rates initially increase as Al content reaches 9 wt.%, which is attributed to a higher fraction of isolated β phases acting as micro-galvanic cathodes. Conversely, further increasing the Al content to 13 wt.% promotes the development of continuous network β phases, which serve as an efficient physical barrier that stops corrosion propagation. Furthermore, the Volta potential difference (VPD) between the α-Mg and β-phase decreases with rising Al concentrations, alleviating galvanic corrosion, further lowering the corrosion rate.

## 1. Introduction

Magnesium (Mg) alloys are receiving great interest as lightweight structural materials in the automotive and aerospace sectors, because of their high specific strength and excellent castability [1,2,3]. Among the Mg alloys, AZ (i.e., Mg–Al–Zn) series alloys represent the most extensively utilized commercial alloy. For mass production, high-pressure die casting is a widely applied manufacturing method due to a good balance between mechanical properties and processability [4,5,6]. However, the disadvantage of Mg alloys to aggressive environments significantly restricts their broader industrial application [7,8,9].

Although commercial AZ series alloys are widely used, their corrosion resistance is still insufficient [10]. To optimize corrosion resistance of AZ series alloys, our team developed the AZ–Ca–Y (AZXW) alloy system. The incorporation of Ca is primarily intended to suppress melt ignition during the casting process via the growth of a dense surface CaO film [10]. Concurrently, Y is introduced to facilitate the removal of Fe impurities, which are detrimental to corrosion resistance [11]. During the melting process, Y combines with Fe to form the Al_8_Fe_4_Y phase and settles down to the bottom. Furthermore, Y drives the precipitation of the Al–Mn–Y phase; because this phase exhibits a lower electrochemical potential than the standard Al–Mn phase, thereby potentially reducing the intensity of micro-galvanic corrosion [11,12]. Therefore, the development of AZXW alloy results in a simultaneous enhancement in both the ignition and corrosion resistance of commercial AZ-series alloy.

The Al content in AZ-based alloys exerts a critical influence on determining their chemical and mechanical properties. Basically, increasing the Al content improves mechanical properties via grain refinement, solid solution strengthening, and precipitation strengthening [12,13,14,15]. The corrosion behavior of AZ series alloys strongly depends on Al content, primarily due to the associated microstructural evolution [16,17,18]. In these alloys, corrosion is predominantly governed by micro-galvanic coupling, where the α-Mg matrix behaves as the anode undergoing anodic dissolution, while the β-Mg_17_Al_12_ phase, possessing a more noble potential, serves as the cathode, facilitating cathodic reaction [16]. Consequently, in terms of corrosion, the Al enrichment can elevate the matrix potential and suppress the anodic reaction [19]. However, the introduction of Al also increases the fraction of β phases, which serve as cathodic sites and intensify the corrosion rate when the β phases are distributed as discrete, isolated particles [17,20,21]. In contrast, when these β phases form a continuous, interconnected network, which acts as a physical barrier, thereby impeding the corrosion propagation [22,23,24].

Cheng. et al. [24] examined the corrosion properties between AZ31 and AZ91, and found that AZ91 exhibits a relatively higher corrosion rate. This phenomenon is rationalized by the higher fraction of β phases, which act as cathodic sites and accelerate micro-galvanic corrosion. However, in Mg–Al binary alloys with varying Al content, a different trend has been reported [17]. Initially, the corrosion rate accelerates with rising Al content owing to the increased fraction of β phases, which enhances galvanic corrosion. With further addition of Al content, the corrosion rate decreases, as the β phases form an interconnected network that provides a t shielding effect, thereby inhibiting corrosion propagation.

The effect of Al concentration on the behavior of AZ-based alloys remains a subject of debate across different alloy systems, as it governs the microstructural evolution and the subsequent corrosion properties [25,26,27,28]. Therefore, this study comprehensively investigated the influence of varying Al concentrations on corrosion properties in a multi-component AZXW alloy. The corrosion resistance was evaluated through weight loss measurement, electrochemical characterization, and SKPFM tests. Consequently, the relationships between microstructural variations and corrosion mechanisms were established.

## 2. Experimental

### 2.1. Specimen Preparation

The high-pressure die-casting (HPDC) AZXW alloys were fabricated using AZ ingots, Mg–20Ca, and Mg–30Y master alloys. The HPDC processing was conducted at MOBASE (Ansan, Republic of Korea) utilizing a hot-chamber die-casting machine with a nominal clamping capacity of 200 tons. The melt and die temperatures were maintained at 660 °C and 260 °C, respectively. Injection of the melt into the die cavity was executed at a continuous pressure of 140 bar, maintaining a plunger speed between 4 and 5 m/s. Four alloy compositions with varying Al contents (6, 9, 11, and 13 wt.%) were produced and designated as AZXW6100, AZXW9100, AZXW11100, and AZXW13100, respectively. The selection of 6 and 9 wt.% Al was directly compared with commercial AZ61 and AZ91 alloys, providing a direct baseline for comparison. Additionally, the 11 and 13 wt.% Al contents were selected to span across the maximum solubility limit of Al in Mg, which stands at 12.7 wt.% at 437 °C. The chemical composition analysis of the alloys was conducted using an optical emission spectrometer (QSN750-II, OBLF, Lüdinghausen, Germany); the resulting data are summarized in Table 1.

### 2.2. Microstructure Characterization

The microstructural examination of the AZXW alloys was performed using an optical microscope (OM, BX51M, OLYMPUS, Tokyo, Japan), Scanning Electron Microscopy (SEM, JSM-7001F, JEOL, Tokyo, Japan) coupled with energy-dispersive X-ray Spectroscopy (EDS). Prior to microstructure observation, the specimen was mechanically ground with SiC emery papers up to 2400 grit, and subsequently polished down to 0.25 μm utilizing a specialized silica suspension to obtain a mirror-like finish. Chemical etching was subsequently performed for 2 s using a picric acid solution (3 g picric acid + 10 mL distilled water + 10 mL acetic acid + 100 mL ethanol). X-ray diffraction (XRD; Smartlab XE, Rigaku, Tokyo, Japan) measurements were performed at a 40 kV operating voltage and a current of 300 mA, utilizing a scan rate of 3°/min over a 2θ range from 20 to 80°.

To ensure accurate compositional analysis of the β phase while minimizing interference from the α-Mg, a specialized specimen preparation technique was employed. After initial polishing according to the aforementioned procedure, the specimens underwent deep etching for approximately 1 min in a picric acid solution. This process selectively dissolved the surrounding α-Mg matrix, effectively protruding the β phase for isolated analysis, as illustrated in Figure S1. This deep-etching approach ensured that the EDS point measurements of the β phase were not influenced by the background signal of the underlying matrix, providing higher analytical precision.

### 2.3. Weight Loss Tests

Weight loss tests were conducted in an aqueous 3.5% NaCl solution at 25 °C for a duration of 10 d, following the ASTM G1-25 standard [29]. After the immersion period, the corrosion products were cleaned using a specific pickling solution composed of CrO_3_ (200 g), Ba(NO_3_)_2_ (20 g), and AgNO_3_ (10 g). The corrosion rates (mm/y) were then calculated from the mass differential of the specimens recorded before and after exposure. Specifically, all weight loss measurements were performed on at least three identical specimens to ensure statistical reliability.

### 2.4. Electrochemical Impedance Spectroscopy

Electrochemical impedance spectroscopy (EIS) measurements were performed using a ZIVE MP1 potentiostat (WonATech, Seoul, Republic of Korea). A conventional three-electrode cell was utilized, including the specimen as the working electrode, an Ag/AgCl as the reference electrode, and a graphite rod as the counter electrode. The tests were performed in a 3.5% NaCl solution at 25 °C after a 72 h exposure. EIS scans were recorded over a frequency range from 100 k Hz to 0.01 Hz with an AC amplitude of ±10 mV at a scan rate of 10 points per decade. The collected EIS data were fitted using ZsimpWin software (AMETEK Scientific Instruments, Oak Ridge, TN, USA).

### 2.5. Scanning Kelvin Probe Force Microscopy

To quantify the micro-scale work function variations and map the VPD between the α-Mg matrix and the second phases, scanning Kelvin probe force microscopy (SKPFM) was performed utilizing an XE-100 system (Park Systems, Gwacheon-si, Gyeonggi-do, Republic of Korea). Specimen preparation involved grinding steps with SiC papers from #600 down to #2400 grit. Final surface was executed via polishing using a colloidal silica suspension to achieve a scratch-free, 0.25 μm mirror finish. Prior to scanning, the samples were ultrasonically cleaned in ethanol and dried under an air stream. The SKPFM data collection was conducted under ambient conditions (25 °C). Surface topography and potential maps were recorded simultaneously across 256 by 256 pixels at a specialized scan rate of 0.35 Hz.

## 3. Results

### 3.1. Microstructure

**Figure 1:**
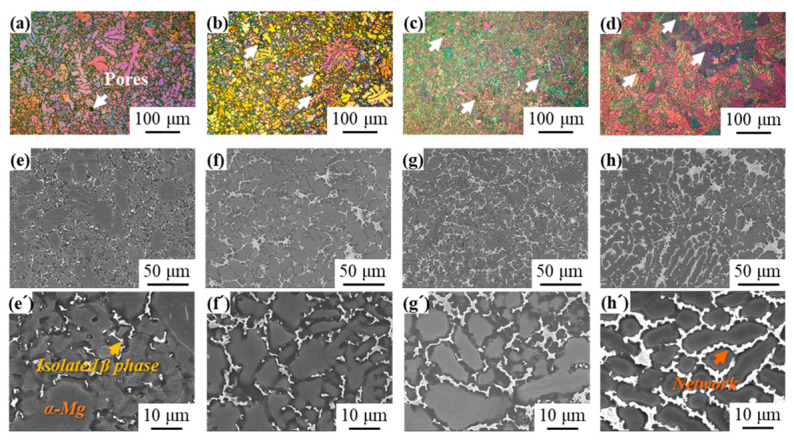
OM and SEM images of AZXW alloys: (**a**,**e**,**e’**) AZXW6100, (**b**,**f**,**f’**) AZXW9100, (**c**,**g**,**g’**) AZXW11100, and (**d**,**h**,**h’**) AZXW13100.

**Figure 2:**
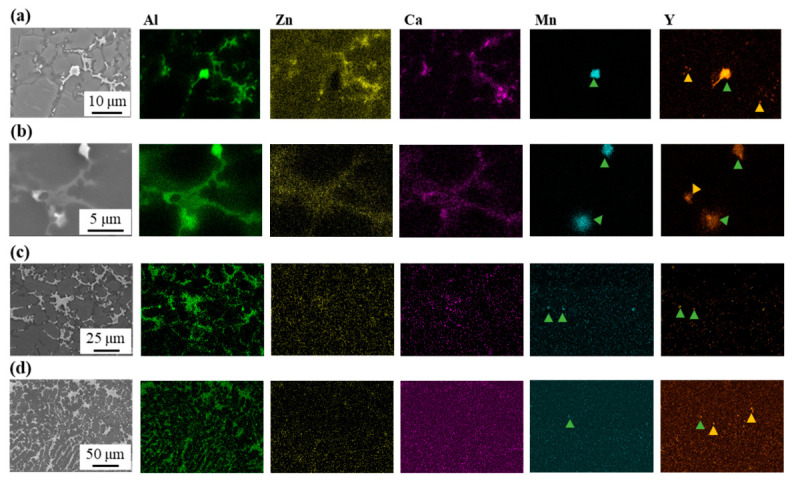
SEM images and corresponding EDS maps for elements Al, Zn, Mn, Ca, and Y: (**a**) AZXW6100, (**b**) AZXW9100, (**c**) AZXW11100, and (**d**) AZXW13100. The green and yellow triangles indicate the Al–Mn–Y and Al–Y phases, respectively.

**Figure 3:**
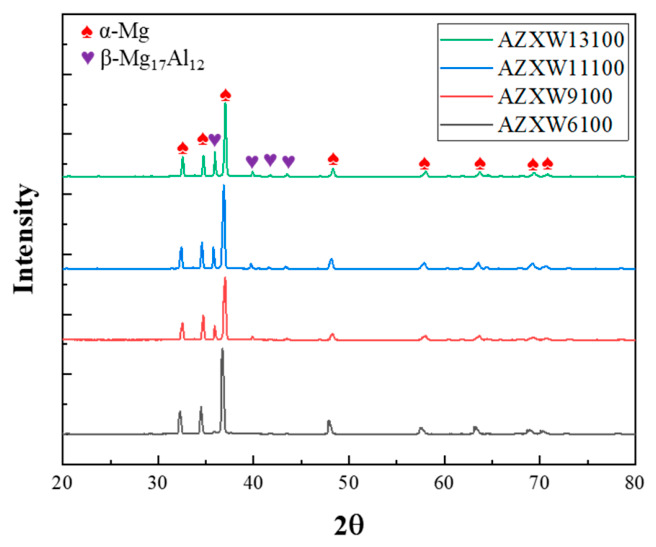
XRD analysis of the AZXW alloys.

**Figure 4:**
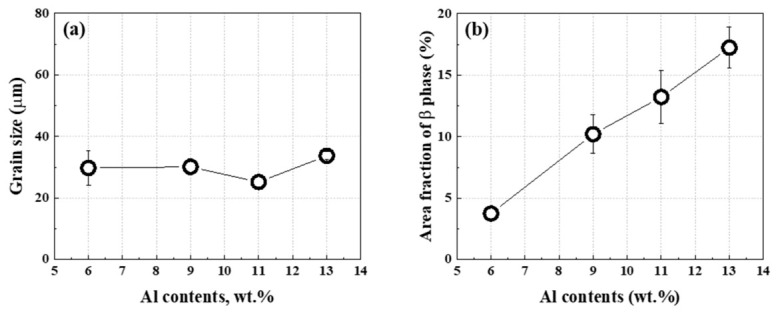
Parameters of AZXW alloys measured from OM and SEM images: (**a**) Grain size and (**b**) Area fraction of β phase.

Figure 1 shows the OM and SEM images of AZXW alloys, and Figure 2 presents the SEM images with their corresponding EDS maps. To further confirm the second phase compositions, XRD analysis is illustrated in Figure 3. The grain sizes (measured via OM) and the area fractions of the β phase (measured from SEM images) are summarized in Figure 4a and Figure 4b, respectively. As shown in Figure 4a, the average grain sizes for AZXW6100, AZXW9100, AZXW11100, and AZXW13100 are 27.23 ± 2.72, 30.11 ± 2.7, 25.17 ± 1.73, and 33.59 ± 1.45 μm, respectively. These results indicate that grain size remains relatively stable despite variations in Al content. Conversely, Figure 4b demonstrates that the β phase fraction enlarged progressively with higher Al concentrations.

The microstructures primarily include the α-Mg (gray areas) and β phases (white areas), as indicated by arrows in Figure 1e’. The β phase segregates preferentially at grain boundaries; as the Al content increases, these β phases develop into the network-like structures, as shown in Figure 1h’. Additionally, Figure 2 confirms the presence of the Al–Y and Al–Mn–Y phases. However, these two phases were not detected in the XRD analysis, because their low volume fractions were below the detection limit of conventional XRD. No significant changes in the area fraction, size, or distribution of these Al-rich phases were observed with an increasing Al content. Therefore, their influence on corrosion behavior was considered negligible. Furthermore, Zn and Ca were found to be primarily partitioned within the β phases.

**Figure 5:**
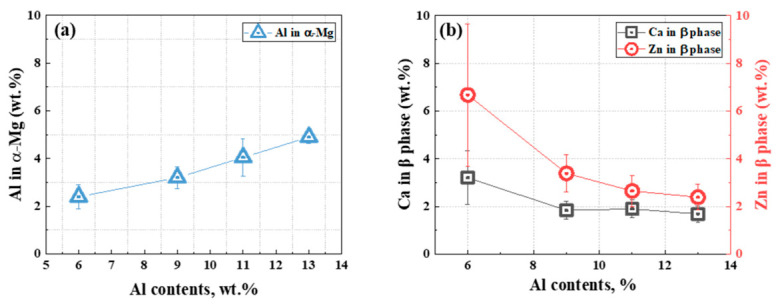
Elemental composition of the α-Mg and β phase of AZXW alloys: (**a**) Al composition in the α-Mg and (**b**) Zn and Ca composition in the β phase, wt.%.

Table 2 summarizes the chemical compositions of both the α-Mg matrix and β phases, as determined by EDS point analysis. While Al and Zn were detected within the α-Mg matrix, the Zn content remained negligible. The β phases mainly consist of Al, with trace amounts of Zn and Ca elements. Figure 5 illustrates the compositional variations in the α-Mg matrix and β phases with respect to Al concentration. As the overall Al content increases, the Al content within the α-Mg matrix also rises. Conversely, the concentration of Zn and Ca within the β phases exhibits a decreasing trend with increasing Al content.

### 3.2. Weight Loss Test and Corrosion Morphology

**Figure 6:**
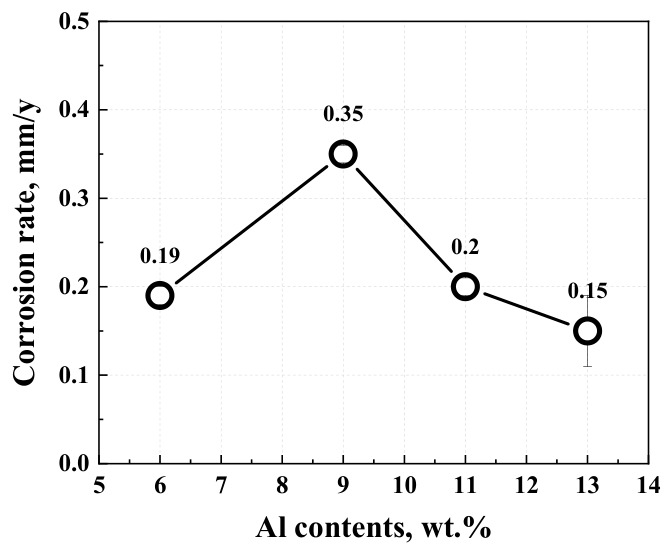
Corrosion rates of AZXW alloys after immersion in a 3.5% NaCl solution for 10 days at 25 °C.

Figure 6 displays the calculated corrosion rates for the various AZXW alloys following 10 d of immersion in a 3.5% NaCl solution. The corrosion rate initially increases from 0.19 ± 0.01 mm/y (6 wt.% Al) to a peak of 0.35 ± 0.01 mm/y (9 wt.% Al). However, as the Al content further increases to 13 wt.%, the corrosion rate declines to 0.15 ± 0.04 mm/y in the AZXW13100 alloy.

**Figure 7:**
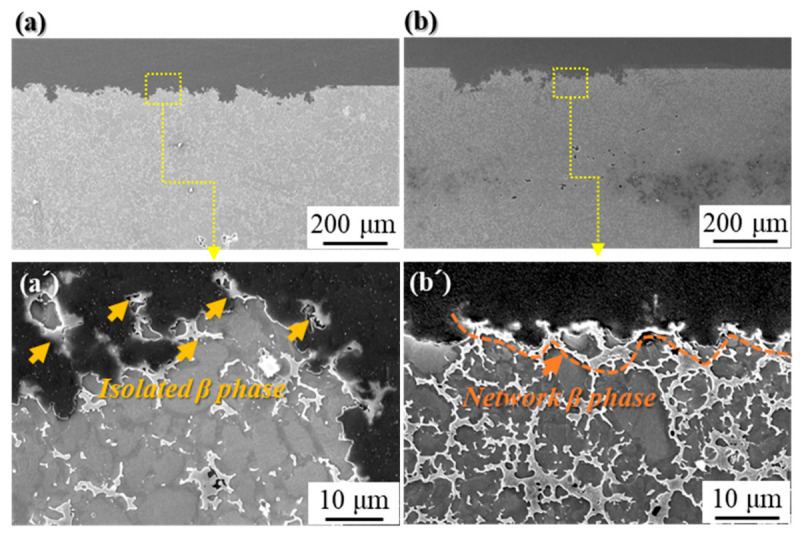
Cross-section morphologies of AZXW alloys after immersion in a 3.5% NaCl solution for 10 days at 25 °C: (**a**,**a’**) AZXW9100 and (**b**,**b’**) AZXW13100.

To investigate the corrosion behavior, cross-section microstructures were observed. Figure 7 illustrates the cross-section microstructures of the AZXW9100 and AZXW13100 alloys after 10 d of immersion. In these alloys, the α-Mg matrix acts as a micro-galvanic anode relative to the β phase, thereby inducing severe micro-galvanic acceleration. In the AZXW9100 alloy, the isolated β phase primarily acts as a cathode, accelerating the dissolution of the matrix. Furthermore, the isolated β phase was detached from the bulk matrix. Conversely, the interconnected β-phase networks of the AZXW13100 alloy remain along the corrosion boundaries. This result reveals that the interconnected β phase displays an effective physical barrier against further corrosion penetration.

### 3.3. Electrochemical Impedance Measurements

**Figure 8:**
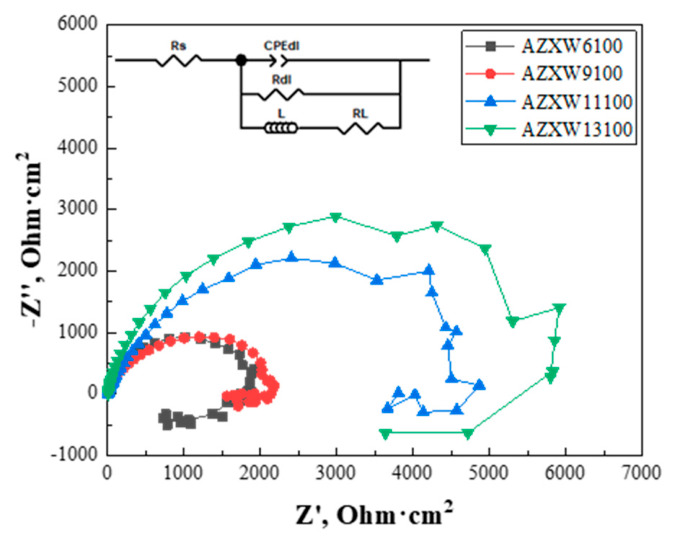
Nyquist plots with fitted equivalent circuit of AZXW alloys were conducted in 3.5% NaCl solution at 25 °C for 72 h.

Figure 8 shows the Nyquist plots with the fitted equivalent circuit for the AZXW alloys with different Al contents after 72 h of immersion. The equivalent circuit is widely utilized for fitting the EIS data of Mg corrosion [18,21,30,31]. Within this equivalent circuit, *R*_s_ represents the solution resistance, CPE_dl_ and *R*_ct_ denote the double-layer capacitance and the charge transfer resistance, respectively. Additionally, L and *R*_L_ represent the inductor and inductive resistance. Because *R*_ct_ is inversely proportional to the corrosion rate, a higher *R*_ct_ value indicates a lower corrosion rate.

The fitted parameters corresponding to the CPE and resistance are summarized in Table 3. The parameter *n* is the CPE exponent; values of 1, 0, and −1 correspond to an ideal capacitor, resistor, and inductor, respectively [32]. The *R*_s_ values across all alloys are similar, while the dimension of the capacitive loops is significantly altered by the Al content. Moving from high to mid frequency, the capacitive loop reflects the double layer and *R*_ct_ [32]. In the low frequency range, an inductive loop is clearly observed, which is attributed to the surface relaxation of adsorbed Mg^+^ intermediates [33,34,35,36]. This relaxation process is attributed to the instability of the Mg(OH)_2_ corrosion products in the NaCl solution. Over the 72 h immersion period, the Mg(OH)_2_ reacted with Cl^−^ ions to form soluble MgCl_2_, leading to localized film breakdown and the subsequent appearance of the inductive loop [32].

## 4. Discussion

### 4.1. Effect of Al Content on Microstructure

Increasing the Al content results in a slightly higher Al concentration within the α-Mg matrix (Table 2) and a greater area fraction of the β phase (Figure 4b). As a consequence of the solid solubility of Al in the α-Mg matrix being restricted at a given temperature, the excess Al precipitated as the β phases, resulting in an increased β-phase fraction [13]. This higher fraction promotes greater connectivity among β phases, ultimately forming the network structures observed in Figure 1h’. These network β phases continuously distribute along the grain boundaries, effectively encompassing the grains.

Table 1 indicates that the Zn content remains relatively constant across all investigated alloys. Therefore, the higher fraction of β phases (Figure 4b) results in a corresponding reduction in Zn concentration within individual β particles (Figure 5b). This behavior can be ascribed to a dilution effect, where the total available Zn is distributed across a larger fraction of β phase. Similarly, the Ca concentration within the β phases exhibits a decreasing trend as the phase fraction increases, suggesting a consistent partitioning behavior for these alloying elements as the Al content rises.

### 4.2. Effect of β Phase Morphology on Corrosion Behavior

The distribution of the β phase is a decisive factor in governing the corrosion resistance of high-Al Mg alloys. Indeed, morphology and area fraction of the β phase are considered critical parameters in determining the overall degradation rate [18,37,38]. Generally, the influence of the β phases on corrosion is twofold: (i) it functions as an efficient electrochemical cathode, thereby accelerating micro-galvanic corrosion of the adjacent α-Mg matrix [39]; and (ii) it can serve as a physical barrier that retards the progression of the dissolution front, leading to its low corrosion rate [18].

In alloys with low Al content, the β phases are spatially isolated (Figure 1). With increasing Al concentration, the fraction of isolated β phases is enhanced. Cross-section morphologies (Figure 7a’) reveal that the α-Mg matrix interfacing with the isolated β phases undergoes preferential dissolution. In this morphology, the β phases serve only as cathodic sites, accelerating micro-galvanic corrosion that penetrates deeply into the unprotected α-Mg matrix, thereby exacerbating overall material degradation. Therefore, the Mg electrode readily undergoes anodic dissolution to form Mg^2+^, yielding relatively low *R*_ct_ in AZXW6100 (1868 Ω∙cm^2^) and AZXW9100 (1783 Ω∙cm^2^) alloys.

In contrast, the corrosion propagation is effectively obstructed by the network β phases, as observed in Figure 7b’. The higher fraction of these phases in high-Al alloys creates a physical shielding effect, suppressing the corrosion propagation, thereby lowering the overall corrosion rate (Figure 6). Consequently, when Al content increases from 6 to 9 wt.%, the corrosion rate rises because the increase in cathodic sites (isolated β phases) outweighs the protective effect of the nascent network. However, upon further increasing the Al content to 13 wt.%, the β phases predominantly form a continuous network that functions as a protective barrier, effectively mitigating the corrosion rate. The β phase fraction of approximately 13.22 ± 2.14% in AZXW11100 alloy serves as a critical threshold. When the β phase fraction exceeds 13.22%, a critical transition from micro-galvanic acceleration to a highly effective barrier effect results in a reduction in corrosion rate (Figure 6). This structural evolution and the corresponding phase fraction metrics are clearly supported by the SEM microstructures in Figure 2 and the quantitative data in Figure 5b, respectively. The increase in fraction isolated β phases accelerates the micro-galvanic corrosion kinetics of the Mg matrix, leading to the lowest *R*_ct_ in AZXW9100 alloy. This network β-phase barrier effectively shields the underlying Mg matrix from continuous exposure to the electrolyte. Consequently, the *R*_ct_ value reaches its maximum (6653 Ω∙cm^2^) in the AZXW13100 alloy, as shown in Figure 8 and Table 3.

### 4.3. Effect of β Phase Composition on Corrosion Resistance

**Figure 9:**
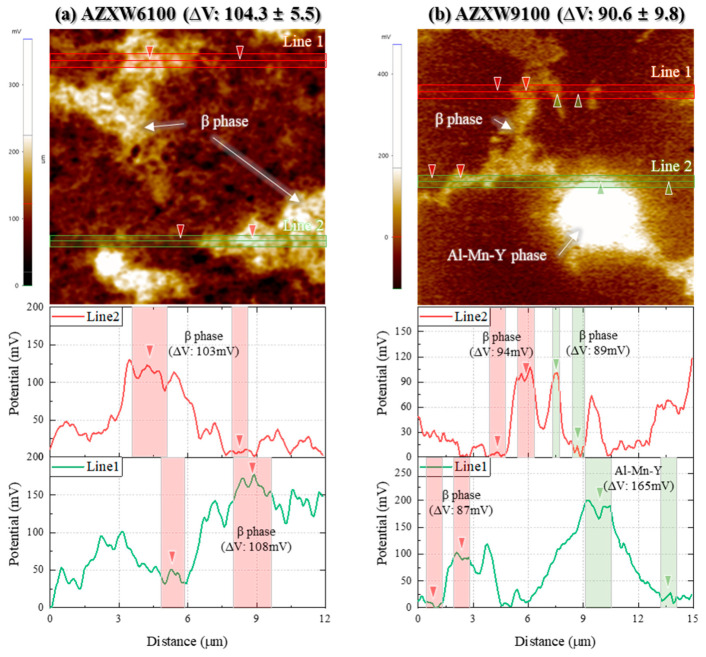

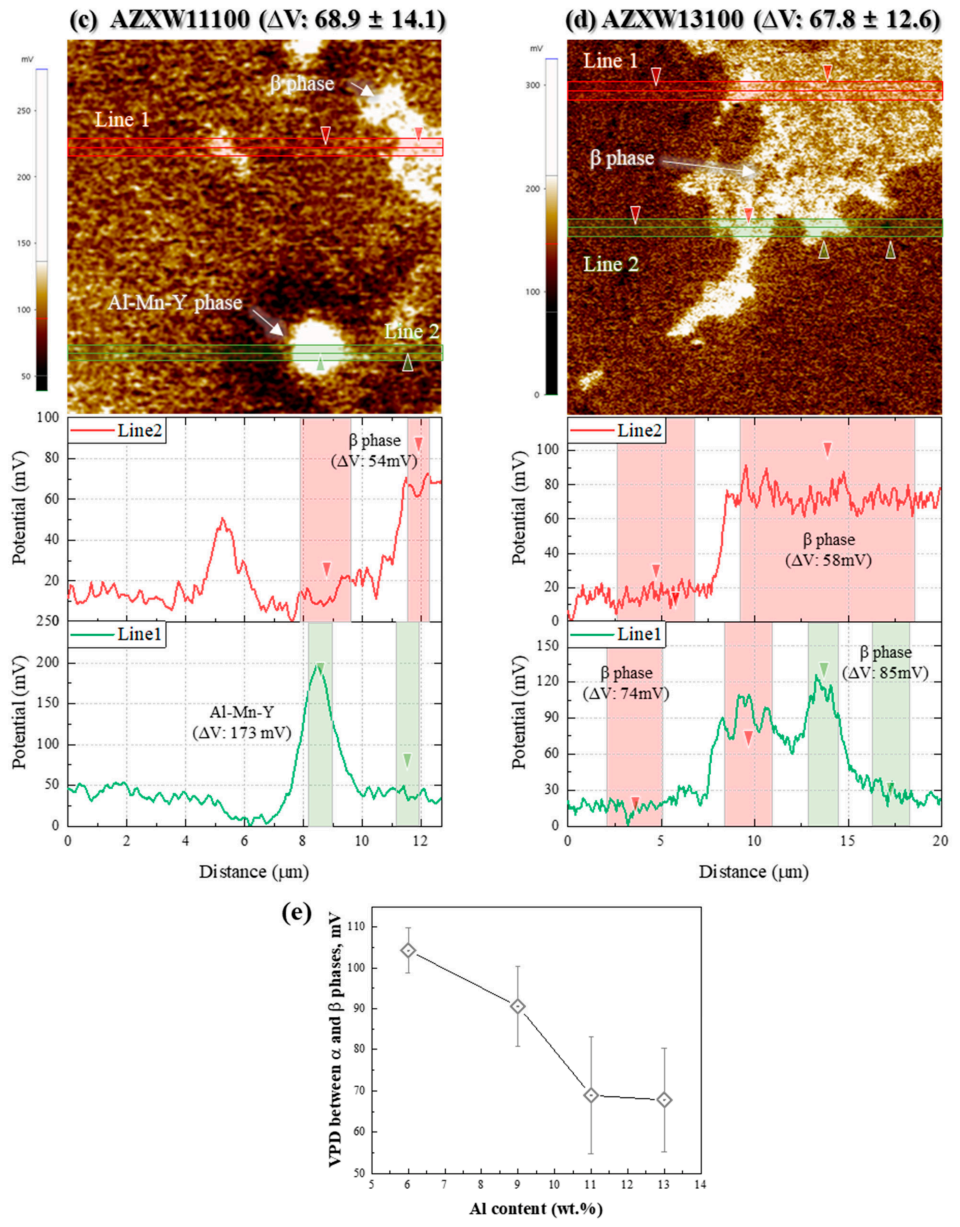
Surface potential maps and profiles analyzed by SKPFM: (**a**) AZXW6100, (**b**) AZXW9100, (**c**) AZXW11100, and (**d**) AZXW13100 alloys, (**e**) VPD plots of all AZXW alloys. The red and green arrows in the figure and graph indicate the same corresponding identical positions.

Micro-galvanic corrosion typically originates within the α-Mg matrix, interfacing with the β phases. Therefore, the VPD between these two phases is a critical factor governing the corrosion behavior. To quantify this, SKPFM measurements were performed on the AZXW9100 and AZXW13100 alloys, serving as representative specimens. Figure 9 shows the VPD maps and plot for AZXW6100, AZXW9100, AZXW11100, and AZXW13100 alloys, with additional results provided in Figure S2 to substantiate the average values. The interfacial VPD between the α-Mg matrix and β phase exhibits a continuous downward trend with increasing Al content, decreasing from 104.3 ± 5.5 mV in the AZXW6100 alloy to 90.6 ± 9.8 mV in the AZXW9100 alloy, and dropping further to 68.9 ± 14.1 and 67.8 mV ± 12.6 mV for the AZXW11100 and AZXW13100 alloys, respectively.

The reduction in the VPD with an increase in the Al contents in the alloys might be associated with compositional alterations within both the constituent α-Mg and β phases. This is expected given the distinct standard electrode potentials of Al (−1.66 V vs. SHE), Ca (−3.8 V vs. SHE) and Zn (−0.76 V vs. SHE) compared to Mg (−2.38 V vs. SHE). As reported by M. Grimm et al. [19], the α-Mg potential changed only marginally with Al concentrations between 2 and 9 wt.%; thus, the influence of matrix Al enrichment on the VPD is believed to be negligible. Furthermore, while the incorporation of Ca into the β phase is documented to decrease the VPD [40], the AZXW alloy with a higher Ca content exhibits a lower VPD. This higher VPD in AZXW alloy may be attributed to Zn concentration within the β phases. Specifically, the dilution of Zn likely shifts the β phase potential, thereby reducing the VPD between the α-Mg matrix and β phase. This Zn dilution within the β phase observed in Figure 4 directly corresponds with the continuous downward trend observed in the VPD plot of Figure 9e, indicating a relationship between the Zn concentration and the resulting VPD. Consequently, as the Al content increases in the AZXW alloys, the declining VPD diminishes the driving force for micro-galvanic corrosion.

**Figure 10:**
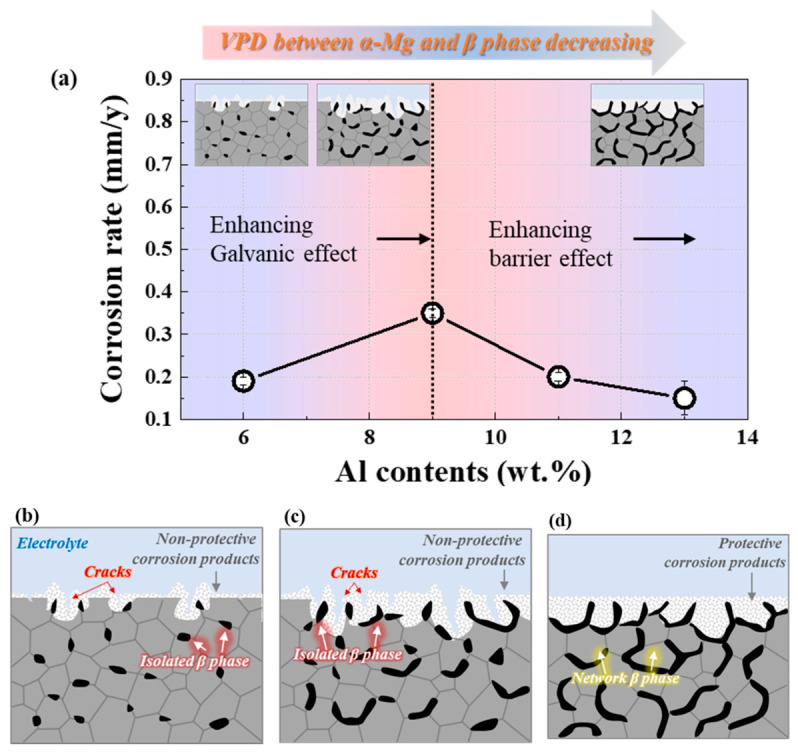
(**a**) Relationship among corrosion rate, VPD, and β-phase morphology as a function of Al content; (**b**–**d**) schematic diagrams illustrating the corrosion mechanisms for (**b**) isolated β-phase with non-protective corrosion products, (**c**) semi-continuous network with non-protective corrosion products, and (**d**) fully continuous network with protective corrosion products.

Figure 10 schematically illustrates the corrosion mechanism of the AZXW alloys in response to Al enrichment. In alloys with a low fraction of β phases, these phases only serve as discrete cathodic sites, accelerating micro-galvanic degradation in the α-Mg matrix (Figure 10b). As the Al content increases up to 9 wt.%, although a nascent network begins to form, a significant number of β phases continue to act as active cathodes (Figure 10c), thereby increasing the overall corrosion rate. However, upon progressive additions of Al up to 13 wt.%, the increased β-phase fraction promotes the formation of networks. In this morphology, the barrier effect outweighs the cathodic acceleration, as the network β phases shield the matrix and prevent the inward propagation of the corrosion front (Figure 10d). Furthermore, a higher alloying Al content leads to an increased concentration of Al dissolved into the α-Mg matrix, as shown in Figure 5a. Previous studies have reported that the enrichment of dissolved Al within the matrix results in the formation of a denser, more compact Al_2_O_3_-containing surface film [17,41,42]. Consequently, the corrosion product becomes more compact in the AZXW13100 alloy, as depicted in Figure 10d. Additionally, high-Al alloy exhibits a lower VPD between the α-Mg and β phases, which effectively suppresses the driving force for micro-galvanic acceleration and further contributes to the overall reduction in the corrosion rate.

### 4.4. Comparison of Corrosion Rates in Mg–Al-Based Alloys

**Figure 11:**
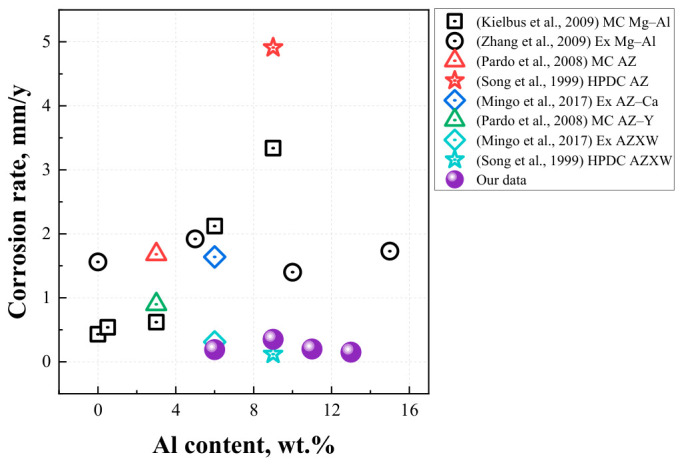
Comparison of corrosion rates in Mg–Al-based alloys with Al contents in the alloys [13,38,39,40,41].

A comprehensive comparison of the corrosion rates of various Mg–Al-based alloys from the literature is summarized in Table 4 and plotted in Figure 11. Overall, the AZXW alloys exhibit significantly lower corrosion rates than both pure Mg and Mg–Al-based alloys. When comparing the processing methodologies among the AZXW alloys, the HPDC alloy demonstrates a superior corrosion resistance relative to their extrusion (Ex) alloys. For mold cast (MC) Mg–Al binary alloys, the corrosion rate continuously increases as the Al content rises from 0 to 9 wt.%. In contrast, Ex Mg–Al binary alloys show a fluctuating trend, where the corrosion rate initially increases up to 5 wt.% Al decreases at 10 wt.% Al, and rises again at 15 wt.% Al. Within our investigated HPDC AZXW alloys, the AZXW9100 alloy represents a critical inflection point, exhibiting the peak corrosion rate before the onset of network shielding.

## 5. Conclusions

This study investigates the corrosion mechanism of AZXW alloys with varying Al content. The average corrosion rates of AZXW alloys following 10 d immersion with 6, 9, 11, and 13 wt.% Al contents are 0.19 ± 0.01, 0.35 ± 0.01, 0.20 ± 0.01, and 0.15 ± 0.04 mm/y, respectively. The highest corrosion rate in AZXW9100 alloy is attributed to the higher fraction of β phase, which predominantly acts as a cathode, accelerating galvanic corrosion. When the Al content exceeds 9 wt.%, the β phases form more continuous networks that serve as barriers against corrosion propagation. Furthermore, SKPFM reveals that high-Al containing alloy exhibits a lower VPD between the α-Mg and β phases, thereby inhibiting galvanic corrosion and reducing the corrosion rate. This study suggests that such a microstructural design offers a viable pathway toward achieving a high-strength, corrosion-resistant Mg alloy system.

## Figures and Tables

**Table 1:** Chemical composition of AZXW alloys, wt.%.

Alloys	Mg	Al	Zn	Mn	Ca	Y	Fe	Ni	Cu	Si
AZXW6100	Bal.	6.139	1.028	0.103	0.403	0.164	0.0030	0.0005	0.0001	0.0208
AZXW9100	Bal.	9.825	0.903	0.103	0.343	0.146	0.0048	0.0005	0.0001	0.0321
AZXW11100	Bal.	11.086	0.842	0.084	0.366	0.123	0.0064	0.0005	0.0001	0.0262
AZXW13100	Bal.	12.516	0.850	0.072	0.364	0.115	0.0094	0.0005	0.0001	0.0242

**Table 2:** Chemical composition of α-Mg and β phases of AZXW alloys, wt.%.

Phase	Elements	AZXW6100	AXW9100	AZXW11100	AZXW13100
α-Mg	Al	2.40 ± 0.51	3.20 ± 0.46	4.05 ± 0.78	4.9 ± 0.26
	Zn	0.15 ± 0.29	0.09 ± 0.13	0.07 ± 0.13	0.07 ± 0.09
	Ca	/	/	/	/
β phase	Al	31.86 ± 2.68	34.30 ± 2.94	33.57 ± 3.00	35.74 ± 2.56
	Zn	6.68 ± 2.98	3.39 ± 0.79	2.65 ± 0.66	2.39 ± 0.54
	Ca	3.21 ± 1.13	1.84 ± 0.37	1.90 ± 0.37	1.69 ± 0.34

**Table 3:** Fitted parameters driven by Nyquist plots of AZXW alloys in 3.5% NaCl solution at 25 °C for 72 h.

Alloy	*R*_s_ (Ω∙cm^2^)	*R*_ct_ (Ω∙cm^2^)	*n*	C_dl_ (F/cm)
AZXW6100	8.46	1868	0.9256	3.12 × 10^−5^
AZXW9100	6.79	1783	0.8317	3.61 × 10^−5^
AZXW11100	2.74	3931	0.8114	3.53 × 10^−5^
AZXW13100	7.64	6653	0.9058	1.22 × 10^−5^

**Table 4:** Comparison of corrosion rates of Mg–Al-based alloys.

References	Alloy	Manufacture	Corrosion Rate, mm/y
[17]	Pure Mg	Extrusion (Ex)	1.56
	Mg–5Al		1.92
	Mg–10Al		1.40
	Mg–15Al		1.73
[43]	Pure Mg	Mold cast (MC)	0.43
	Mg–0.5Al		0.54
	Mg–3Al		0.62
	Mg–6Al		2.12
	Mg–9Al		3.34
[44]	AZXW9100	High-pressure die-cast (HPDC)	0.12
	AZ91		4.91
[45]	AZXW6100	Extrusion (Ex)	0.31
	AZ61–Ca		1.84
[46]	AZ31	Mold cast (MC)	1.68
	AZ31–Y		0.9
Our data	AZXW6100	High-pressure die-cast (HPDC)	0.19
	AZXW9100		0.35
	AZXW11100		0.20
	AZXW13100		0.15

## Data Availability

The original contributions presented in this study are included in the article/Supplementary Material. Further inquiries can be directed to the corresponding authors.

## Supplementary Materials

The following supporting information can be downloaded at: https://www.mdpi.com/article/10.3390/ma19132760/s1.
