# Supplementary material for: Effect of Al Content on Corrosion Properties in Die-Cast AZXW Alloys
Source: Materials (Basel). 2026 Jun 29;19(13):2760. doi: 10.3390/ma19132760 (PMC13362837; doi:10.3390/ma19132760)
Supplement: Supplementary file 1 [file materials-19-02760-s001.zip › materials-4359923-supplementary.pdf]

Effect of Al Content on Corrosion Properties in Die-Cast AZXW Alloys

Hongxiu Liu [1,2], Bong-Sun You [2], Jun-Ho Bae [2,*] and Jae-Yeon Kim [2,*]

[1] School of Materials Science and Engineering, Changwon National University, 20 Changwondaehak-ro, Uichang-gu, Changwon 51140, Gyeongsangnam-do, Republic of Korea; halolhx@kims.re.kr
[2] Lightweight Materials Research Division, Korea Institute of Materials Science, 797 Changwondae-ro, Seongsan-gu, Changwon 51508, Gyeongsangnam-do, Republic of Korea; bsyou@kims.re.kr
* Correspondence: jhbae@kims.re.kr (J.-H.B.), jaeyeonkim@kims.re.kr (J.-Y.K.)

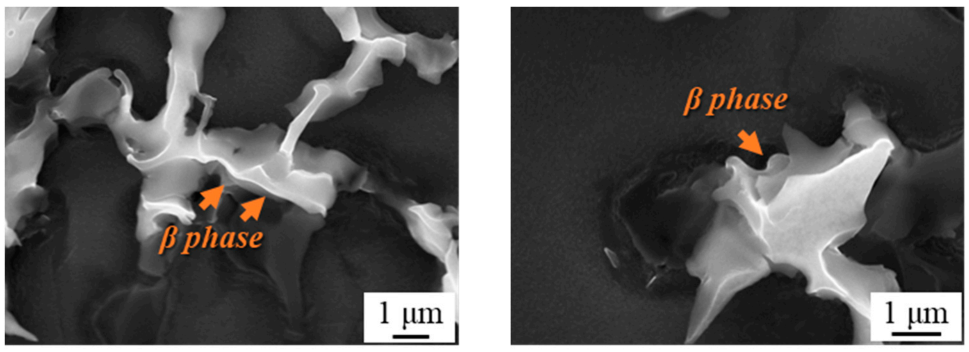

Figure S1. Micrographs of the β phase following deliberate over etching.

(a) AZXW6100

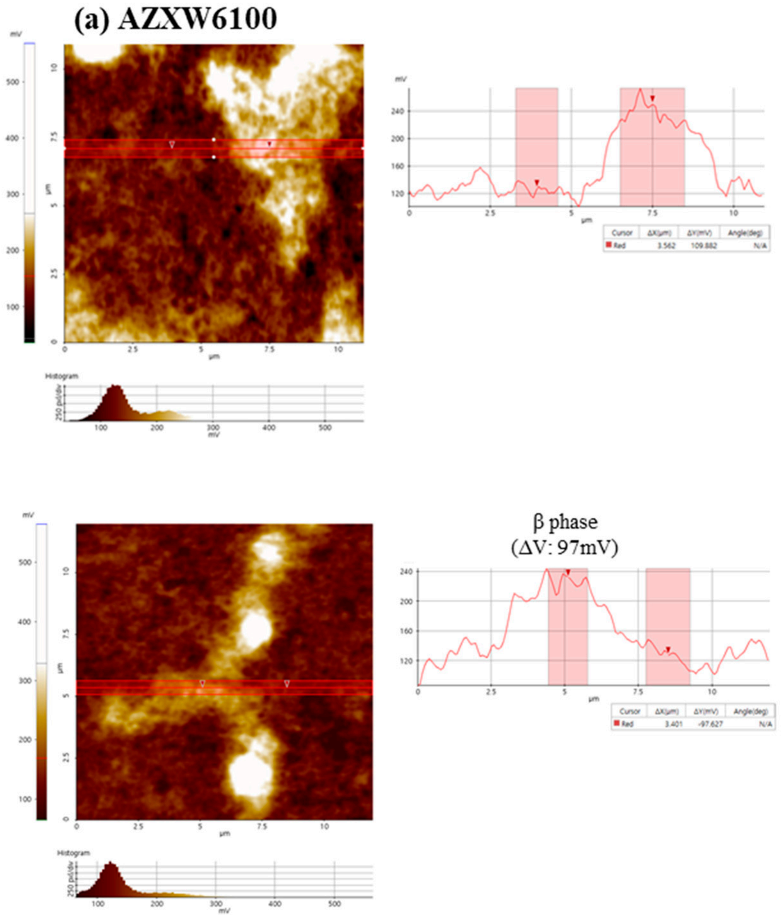

(b) AZXW9100

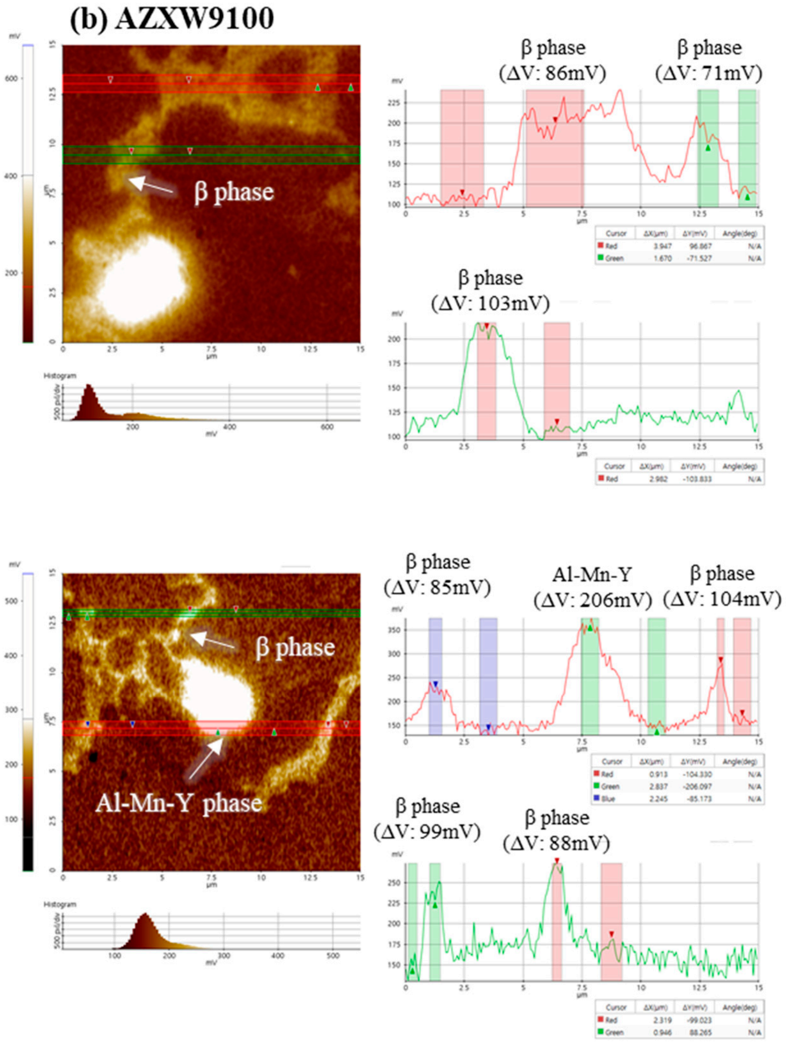
β phase
β phase
(ΔV: 86mV)
β phase
(ΔV: 71mV)
β phase
(ΔV: 103mV)
β phase
Al-Mn-Y phase
β phase
(ΔV: 85mV)
Al-Mn-Y
(ΔV: 206mV)
β phase
(ΔV: 104mV)
β phase
(ΔV: 99mV)
β phase
(ΔV: 88mV)
Histogram

(c) AZXW11100

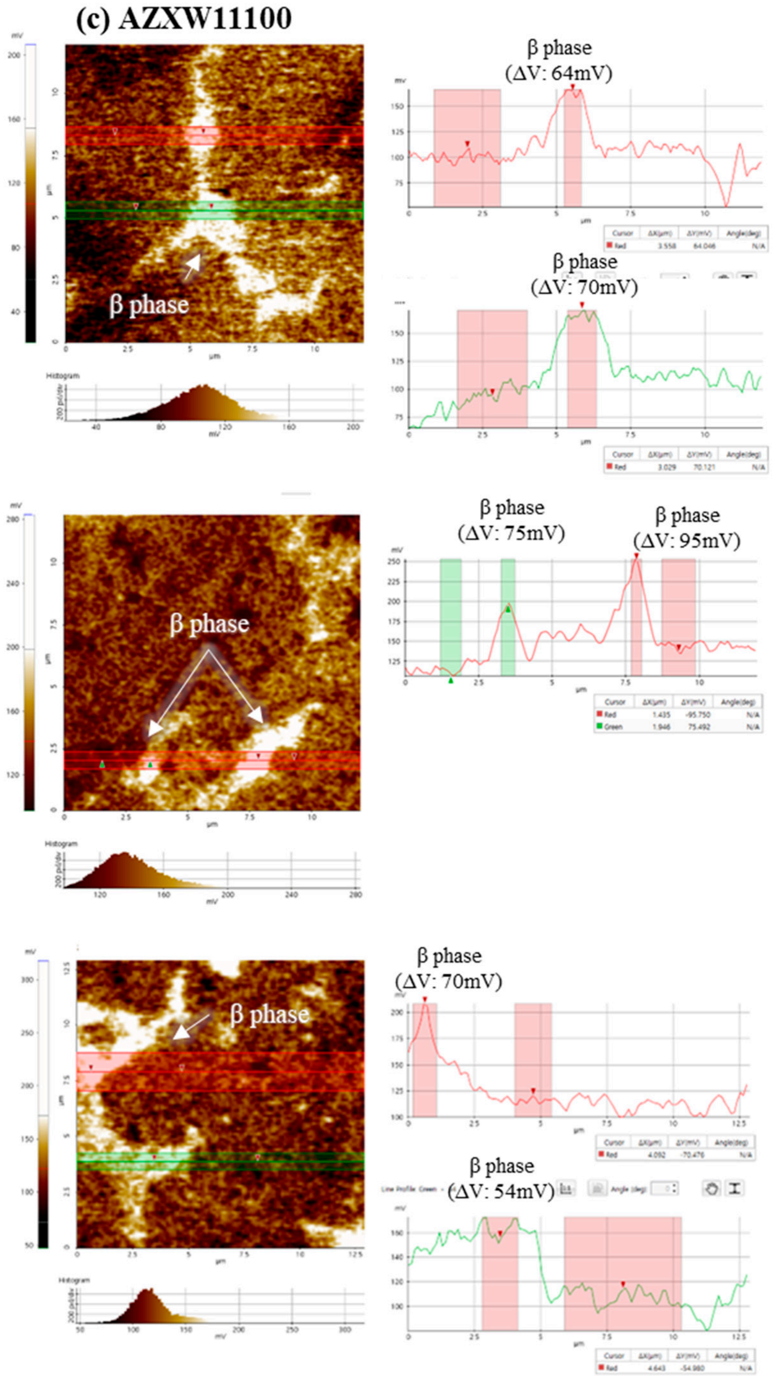
β phase
β phase
(ΔV: 64mV)
β phase
(ΔV: 70mV)
β phase
β phase
(ΔV: 75mV)
β phase
(ΔV: 95mV)
β phase
β phase
(ΔV: 70mV)
β phase
(ΔV: 54mV)

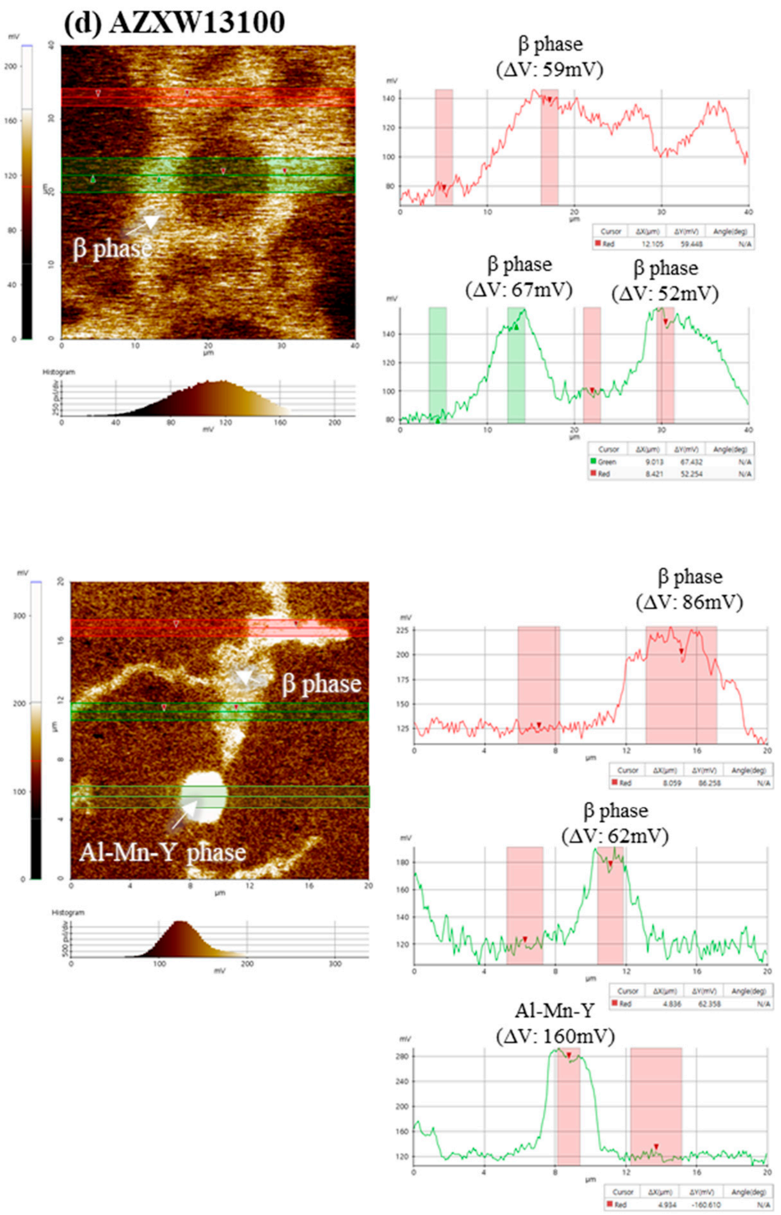

Figure S2. Surface potential maps and profiles analyzed by SKPFM: (a) AZXW6100, (b) AZXW9100, (c) AZXW11100, and (d) AZXW13100.
